# Bioassay-Guided Isolation of Anti-Alzheimer Active Components from the Aerial Parts of *Hedyotis diffusa* and Simultaneous Analysis for Marker Compounds

**DOI:** 10.3390/molecules25245867

**Published:** 2020-12-11

**Authors:** Jun Hee Park, Wan Kyunn Whang

**Affiliations:** Department of Global Innovative Drug, Graduate School, College of Pharmacy, Chung-Ang University, Heukseok-dong, Dongjak-gu, Seoul 151-756, Korea; pjh8255@naver.com

**Keywords:** *Hedyotis diffusa*, bioassay-guided isolation, iridoid glycoside, flavonol glycoside, Alzheimer’s disease, simultaneous analysis

## Abstract

Previous studies have reported that *Hedyotis diffusa* Willdenow extract shows various biological activities on cerebropathia, such as neuroprotection and short-term memory enhancement. However, there has been a lack of studies on the inhibitory activity on neurodegenerative diseases such as Alzheimer’s disease (AD) through enzyme assays of *H. diffusa*. Therefore, *H. diffusa* extract and fractions were evaluated for their inhibitory effects through assays of enzymes related to AD, including acetylcholinesterase (AChE) and butyrylcholinesterase (BChE), and β-site amyloid precursor protein cleaving enzyme 1 (BACE1), and on the formation of advanced glycation end-product (AGE). In this study, ten bioactive compounds, including nine iridoid glycosides **1**–**9** and one flavonol glycoside **10**, were isolated from the ethyl acetate and *n*-butanol fractions of *H. diffusa* using a bioassay-guided approach. Compound **10** was the strongest inhibitor of cholinesterase, BACE1, and the formation of AGEs of all isolated compounds, while compound **5** had the lowest inhibitory activity. Compounds **3**, **6**, and **9** exhibited better inhibitory activity than other compounds on AChE, and two pairs of diastereomeric iridoid glycoside structures (compounds **4**, **8**, and **6**, **7**) showed higher inhibitory activity than others on BChE. In the BACE1 inhibitory assay, compounds **1**–**3** were good inhibitors, and compound **10** showed higher inhibitory activity than quercetin, the positive control. Moreover, compounds **1** and **3** were stronger inhibitors of the formation of AGE than aminoguanidine (AMG), the positive control. In conclusion, this study is significant since it demonstrated that the potential inhibitory activity of *H. diffusa* on enzymes related to AD and showed the potential use for further study as a natural medicine for AD treatment on the basis of the bioactive components isolated from *H. diffusa*.

## 1. Introduction

*Hedyotis diffusa* Willdenow, a member of the Rubiaceae family, is mainly distributed in tropical and sub-tropical Asia, especially in China, Japan, and Indonesia. It has been used to treat appendicitis, dysentery, carbuncles, furuncles, and snake bites in Korea and China’s folk medicine for a long time [1]. Furthermore, previous studies have reported that *H. diffusa* has various biological activities, including neuroprotection [2], short-term memory enhancement [3], anti-oxidant [4], anticancer [5,6], anti-inflammatory [7], antitumor [8], antibacterial [9], and hepatoprotection effects [10]. In addition, various compounds have been isolated from *H. diffusa,* including iridoid glycosides, flavonol glycosides, triterpenoids, flavonoids, anthraquinones, and phenolic acids, such as *E*-6-*O*-*p*-coumaroyl scandoside methyl ester, *E*-6-*O*-*p*-methoxycinnamoyl scandoside methyl ester, *E*-6-*O*-feruloyl scandoside methyl ester, asperuloside, diffusoside A and B, β-sitosterol-3-*O*-β-d-glucoside, quercetin-3-*O*-[2″-*O*-(6‴-*O*-*E*-feruloyl)-β-d-glucopyranosyl]-β-d-glucopyranoside, quercetin-3-*O*-[2″-*O*-(6‴-*O*-*E*-feruloyl)-β-d-glucopyranosyl]-β-d-galactopyranoside, quercetin-3-*O*-[2″-*O*-(6‴-*O*-*E*-sinapoyl)-β-d-glucopyranosyl]-β-d-glucopyranoside, quercetin-3-*O*-sophoroside, *p*-coumaric acid, ferulic acid and ursolic acid [11,12,13,14,15,16,17].

Approximately 17 species of the *Hedyotis* herba genus are used as herbal medicines. In the present study, *H. diffusa* Willdenow has been used as a representative herbal medicine; however, it can often be confused for *H. corymbosa* Lamark owing to their similar external appearance. It is necessary to identify a specific potential biomarker that makes it possible to distinguish *H. diffusa* from counterfeits.

Alzheimer’s disease (AD) is the most frequent cause of dementia in elderly people and is a progressive and irreversible neurodegenerative disorder that results in gradual degradation of cognitive function, memory impairment, and altered behavior, including delusions, paranoid disorders, loss of social appropriateness, and eventually death [18,19,20,21]. Based on decades of research and experience, several changes have been made in the clinical criteria for the diagnosis of AD. Recently, the two most promising hypotheses, including the cholinergic hypothesis and the amyloid hypothesis, were proposed although the exact mechanisms of AD pathogenesis remain unclear [22,23,24,25]. The ‘cholinergic hypothesis’ was the basis for the development of synaptic treatment designed to maintain the activity of the surviving cholinergic system. Biomarkers for cholinergic neurons, such as acetylcholinesterase (AChE) and butyrylcholinesterase (BChE), are the enzymes involved in the synthesis and degeneration of acetylcholine (ACh) and butyrylcholine (BCh), respectively [26,27]. Hydrolysis of the two neurotransmitters, ACh and BCh by AChE and BChE, respectively, results in the progression of AD. The ‘amyloid hypothesis’ proposed that accumulation of β-amyloid peptide (Aβ) in the brain causes the pathogenesis of AD. The abnormal processing of Aβ, which is the result of altered production of Aβ by γ-secretase and amyloid precursor protein (APP) cleavage by β-secretase, or impaired Aβ clearance mechanisms, is one of the factors responsible for progression of AD [28,29]. Since β-secretase initiates Aβ processing, BACE1 inhibition (to prevent the accumulation of Aβ) is considered as one of the treatment strategies against AD [30]. In addition, a previous study showed that accumulation of advanced glycation end-products (AGEs) in the brain is characteristic of aging and deterioration, especially in AD. Increasing AGE levels are neuropathological and biochemical features of AD; they contribute to extensive protein crosslinking (β-amyloid) and neuronal cell death [31]. Therefore, targeting cholinesterases (AChE and BChE), BACE1, and the formation of AGE represents a reasonable therapeutic approach for AD.

Several synthetic drugs have been used as cholinesterase inhibitors, such as tacrine, galantamine, donepezil, and rivastigmine, however, they are associated with adverse side effects, such as nausea, vomiting, gastrointestinal disturbances and poor bioavailability [32]. Because of these side effects, there is an increased need for pharmacological research on natural products such as *H. diffusa*. In this study, we aimed to evaluate the potential efficacy of the extracts, fractions, and compounds isolated from *H. diffusa* against AD, that is, against cholinesterase, BACE1, and AGEs formation. In addition, we aimed to identify biomarkers to help distinguish *H. diffusa* from *H. corymbosa*.

## 2. Results

### 2.1. Structural Identification of Compounds ***1**–**10*** Isolated from H. diffusa

**Figure 1:**
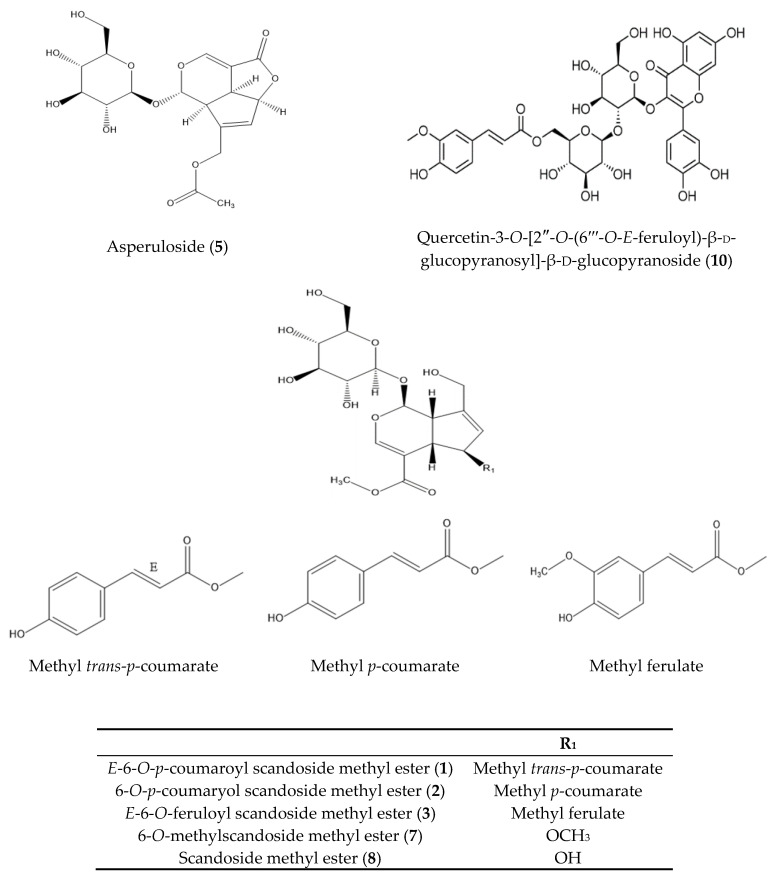

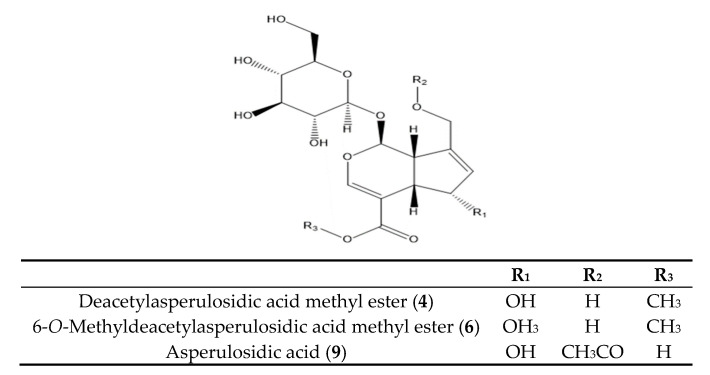
Structures of compounds **1**–**10**.

**Figure 2:**
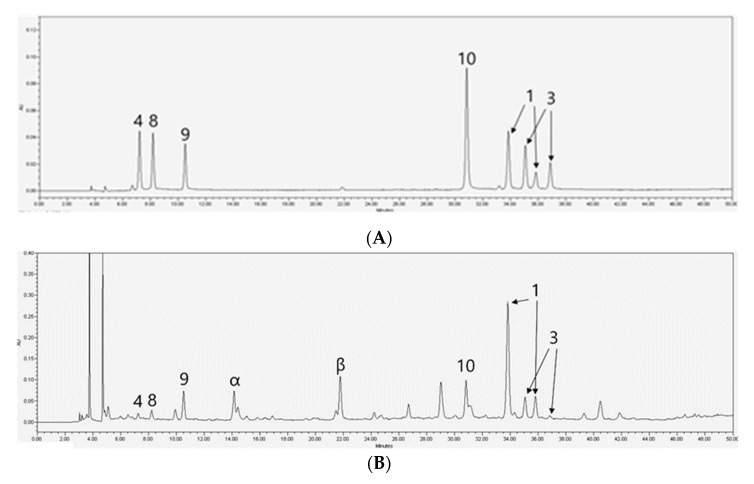

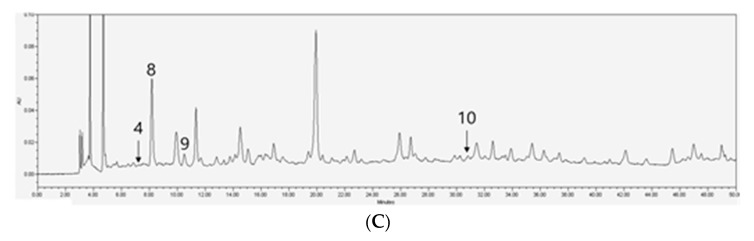
HPLC chromatograms of standard mixture (**A**), *H. diffusa* extract (**B**) and *H. corymbosa* extract (**C**).

**Figure 3:**
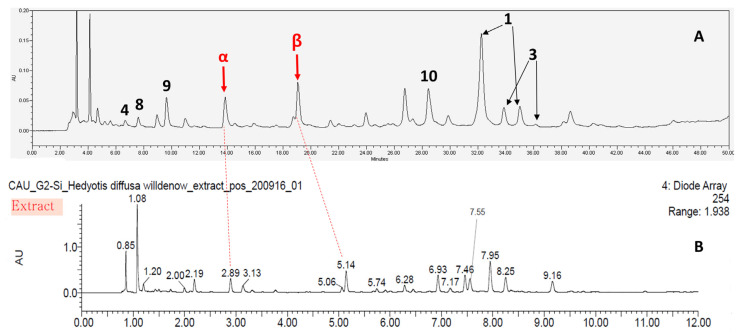
Chromatogram (HPLC) of *H. diffusa* extract (**A**) and UPLC connected QTOF-MS chromatogram of *H. diffusa* extract (**B**).

According to the bioassay-guided isolation method, chromatographic separation of hexane (Hx), dichloromethane (DCM), ethyl acetate (EA), *n*-butanol (BuOH), and distilled water fractions from *H. diffusa* was performed. Finally, nine iridoids **1**–**9** and one flavonol glycoside **10** were isolated. Compounds **1**–**10** isolated from *H. diffusa* were identified as *E*-6-*O*-*p*-coumaroyl scandoside methyl ester (**1**) [11], 6-*O*-*p*-coumaroyl scandoside methyl ester (**2**) [12], *E*-6-*O*-feruloyl scandoside methyl ester (**3**) [11], deacetylasperulosidic acid methyl ester (**4**) [33], asperuloside (**5**) [34], 6-*O*-Methyldeacetylasperulosidic acid methyl ester (**6**) [35], 6-*O*-Methylscandoside methyl ester (**7**) [35], scandoside methyl ester (**8**) [36], asperulosidic acid (**9**) [36], and quercetin-3-*O*-[2″-*O*-(6‴-*O*-*E*-feruloyl)-β-d-glucopyranosyl]-β-d-glucopyranoside (**10**) [17], respectively, based on comparison with spectroscopic data (^1^H-NMR, ^13^C-NMR, and MS) available in the literature (Figure 1). Detailed MS data, such as *m*/*z* data and retention time of each compound, are shown in Table 1. The observed mass value accuracy of compounds **1**–**10** was within 5 ppm, suggesting that the results were reliable. HPLC analysis was performed to determine the major components of *H. diffusa* extract after identifying compounds **1**–**10** (Figure 2). For identifying what compounds two peaks (**α** and **β**) are, a UHPLC-ESI/QTOF-MS was used, and compounds **α** and **β** were identified as asperuloside, and quercetin-3-*O*-sophoroside (Figure 3 and Table 2). In particular, in the chromatogram of *H. diffusa*, each peaks of compounds **1** and **3** were separated by two parts. Since the C-7′ and C-8′ of compounds **1** and **3** exist in *trans* (*E*) and *cis* (*Z*), they are expected to exist in the form (*E*)-6-*O*-*p*-coumaroyl scandoside methyl ester and (*Z*)-6-*O*-*p*-coumaroyl scandoside methyl ester, and (*E*)-6-*O*-feruloyl scandoside methyl ester and (*Z*)-6-*O*-feruloyl scandoside methyl ester, respectively. Previous studies revealed that (*E*)- and (*Z*)-configurations are interconvertible in the presence of heat or light energy [37].

### 2.2. Inhibitory Activities of the Extract and Fractions Obtained from H. diffusa against Cholinesterase (AChE and BChE), BACE1, and the Formation of AGE

In this study, we investigated the inhibitory activities of the *H. diffusa* extract and fractions on cholinesterase, BACE1, and the formation of AGE to demonstrate the efficacy of *H. diffusa* against AD. The results are summarized in Table 3.

The *H. diffusa* extract remarkably inhibited AChE and BChE activity (IC_50_ of 102.01 ± 4.90 and 99.79 ± 4.52 μg/mL, respectively). In the ChE inhibitory assay, the EA and BuOH fractions exhibited the highest inhibitory activity (IC_50_ values of 25.98 ± 3.07 and 1.15 ± 0.32 μg/mL, respectively). Similarly, in the BACE1 inhibitory assay, the EA and BuOH fractions were found to show inhibitory activity much stronger than that of the *H. diffusa* extract and other fractions (IC_50_ of 14.84 ± 1.24 and 26.92 ± 3.48 μg/mL, respectively). In addition, the EA fraction (IC_50_ of 99.32 ± 1.31 μg/mL) showed the strongest inhibitory activity against the formation of AGE followed by the BuOH fraction (IC_50_ of 109.27 ± 5.76 μg/mL).

In summary, the *H. diffusa* extract was an effective inhibitor of AChE, BChE, BACE1, and the formation of AGE. In all assays, the EA and BuOH fractions showed stronger inhibitory activity than the other fractions. In contrast, the DCM and water fractions possessed slight or no potential inhibitory activity.

### 2.3. Inhibitory Activities of Compounds ***1**–**10*** Isolated from H. diffusa against ChE (AChE and BChE), BACE1, and the Formation of AGE

Compounds **3**, **6**, and **9** were much stronger AChE inhibitors than other compounds, except compound **10**. Moreover, this study revealed the following relationships between the iridoid glycoside structure and AChE inhibitory activity: (1) the iridoid glycoside with methyl ferulate on C-6 (compound **3**) was much more active than those with a methyl *trans*-*p*-coumarate and methyl *p*-coumarate substitutent on C-6 (compounds **1** and **2**); (2) iridoid glycosides with a substituted hydroxyl group at the C-6 position (compounds **4** and **8**), which are diastereomers, showed mild activity with IC_50_ values of 172.26 ± 20.55 and 157.68 ± 13.18 μM, respectively; (3) iridoid glycosides with a substituted methoxy group at the C-6 position (compounds **6** and **7**), which are diastereomers, had completely opposite activity. Although compound **6** significantly inhibited AChE, with an IC_50_ value of 81.06 ± 5.58 μM, compound **7** did not inhibit AChE. (4) The iridoid glycoside with a substituted acetyl group on C-10 (compound **9**), which had an IC_50_ value of 68.34 ± 5.11 μM, exhibited the strongest inhibitory activity among the iridoid glycoside compounds.

With respect to BChE inhibitory activity, two kinds of diastereomers among the iridoid glycosides (compounds **4**, **8** and **6**, **7**) showed higher inhibitory activity than others. Furthermore, among compounds **1**–**3**, derivatives of methyl *p*-coumarate at the C-6 position (compound **2**) was more active than the derivatives of methyl *trans*-*p*-coumarate and methyl ferulate (compounds **1** and **3**). Although compound **1**, **2**, **4** and **8** showed mild activity on AChE, these compounds had effective inhibitory activities on BChE.

In the BACE1 inhibitory assay, compound **10** was more effective than quercetin (positive control) and had the highest inhibitory activity against BACE1. Among the isolated compounds, only the iridoid glycosides with methyl *trans*-*p*-coumarate, methyl *p*-coumarate, and methyl ferulate at the C-6 position (compounds **1**, **2**, and **3**) showed considerable inhibitory activity, in that order. Other compounds, except compounds **1**–**3**, had low inhibitory activity against BACE1 among the iridoid glycoside compounds.

Compounds **1** and **3** were stronger inhibitors of the formation of AGE than AMG (positive control). This study suggested the following structural features for inhibition of AGE formation by iridoid glycosides: iridoid glycosides with derivatives of methyl *trans*-*p*-coumarate, methyl *p*-coumarate, and methyl ferulate (compounds **1**–**3**) were much stronger than other compounds, except compound **10**. Compound **4**–**9** showed low or no activity.

In particular, compound **5** had considerably low inhibitory activity against AChE, BChE, BACE1, and the formation of AGE with IC_50_ values of 258.81 ± 7.48, >500, >500, and >1000 μM, respectively. The flavonol glycoside compound (compound **10**) showed significantly high inhibitory activities against AChE, BChE, BACE1, and the formation of AGE with IC_50_ values of 46.22 ± 1.59, 13.77 ± 0.37, 4.49 ± 1.86, and 2.71 ± 0.06 μM, respectively. The results are summarized in Table 4.

### 2.4. Simultaneous Quantitative HPLC Analysis of Six Bioactive Components in H. diffusa and H. corymbosa

**Figure 4:**
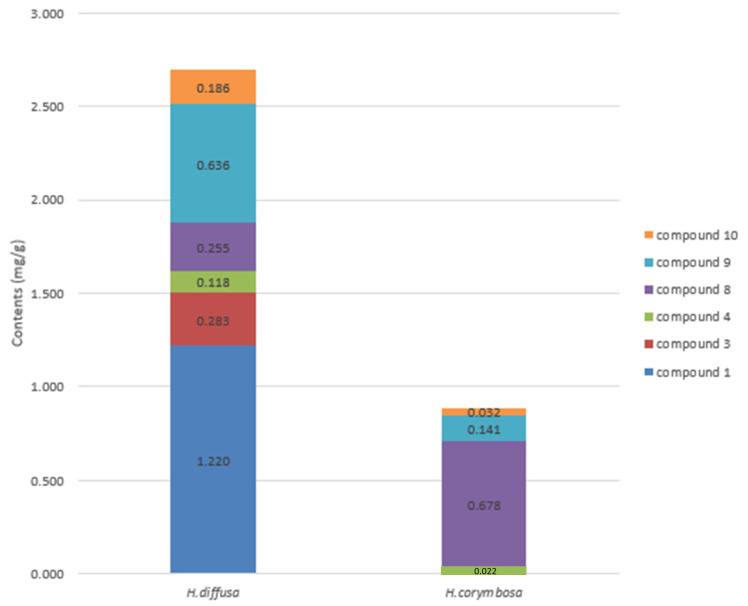
Quantity of major components in *Hedyotis diffusa* and *Hedyotis corymbosa*.

An HPLC analysis of *H. diffusa* and *H. corymbosa* extracts was performed for the quantitative evaluation of the bioactive components (Figure 2). After screening the collected samples of *H. diffusa*, compounds **1** and **9** were identified as the first and second major compounds, respectively, of *H. diffusa* extract. The six bioactive components (**1**, **3**, **4**, **8**, **9**, and **10**) from *H. diffusa* exhibited considerably strong inhibitory activity in AD assays. To optimize the extraction efficiency, samples were extracted by altering the extraction solvent, solvent ratio, and time (Table 5). Among these different extraction times and solvent compositions, the sample extracted after 90 min and using 70% methanol (as solvent) contained the highest amount of the six marker compounds. Furthermore, comparing the two chromatograms of *H. diffusa* and *H. corymbosa*, the major components, compounds **1** and **3**, of the former were not present in the latter (Figure 4). To distinguish between *H. diffusa* and *H. corymbosa*, we discovered, using the developed simultaneous analysis method, that compounds **1** (*E*-6-*O*-*p*-coumaroyl scandoside methyl ester) and **3** (*E*-6-*O*-feruloyl scandoside methyl ester) can be potential biomarkers. The quantity of all compounds, except compound **8** (Scandoside methyl ester), was much higher in *H. diffusa* than in *H. corymbosa* (Figure 4). In *H. diffusa* extract, the quantities of the major bioactive compounds **1**, **3**, and **10** were much higher than those in *H. corymbosa*. These results suggested that simultaneous quantitative HPLC analysis of these six bioactive compounds can be used to obtain quality control standards of *H. diffusa*.

## 3. Materials and Methods

### 3.1. Plant Materials

The aerial parts of *H. diffusa* and *H. corymbosa* were purchased from the Kyung-Dong market, Seoul, Korea, and collected from Busan, Korea, respectively. Prof. Whang Wan Kyunn (College of Pharmacy, Chung-Ang University, Korea) authenticated *H. diffusa* and *H. corymbosa*.

### 3.2. Equipment and Reagents

Methanol (MeOH), ethanol (EtOH), *n*-hexane (Hx), dichloromethane (DCM), ethyl acetate (EA), *n*-butanol (BuOH) (Samchun Pure Chemical, Pyeongtaek, Gyeonggi, Korea) and distilled water were used for extraction, fractionation, and open column chromatography. Open column chromatography used silica gel 60 (40–63 μm; Merck, Darmstadt, Germany), Sephadex LH-20 (25–100 μm; Pharmacia, Stockholm, Sweden), MCI CHP 20P (Supelco, St. Louis, MO, USA), and octadecyl-silica (ODS) gel (400–500 mesh; Waters, Milford, MA, USA). Methanol-*d*_4_ (CD_3_OD) and dimethyl sulfoxide-*d*_6_ (DMSO-*d*_6_) were used for NMR analysis. The molecular weight was determined by ultra-high-performance liquid chromatography (UPLC) and high-resolution mass spectrometry (HRMS) by connecting an electrospray ionization hybrid linear trap-quadruple-Orbitrap MS system (ESI/LTQ-Orbitrap) to an Ultimate 3000 rapid separation liquid chromatography (RSLC) system (Thermo, Darmstadt, Germany). A TECAN Sunrise microplate reader and Infinite F200 pro (Männedorf, Zürich, Switzerland) were used for absorbance and fluorescence, respectively. High performance liquid chromatography (HPLC) was performed using Waters 2695 system pump and Waters 996 Photodiode array detector (Waters, Milford, MA, USA); data was extracted with Empower Pro 2.0 software. The separation column was a Waters Kromasil C_18_ column (4.6 × 250 mm, 5 μm). HPLC-grade solvents (e.g., acetonitrile and distilled water (H_2_O)) were purchased from J. T. Baker^®^ (Phillipsburg, PA, USA). HPLC-grade acetic acid was purchased from Fisher Chemical (Janssen Pharmaceuticalaan, Geel, Belgium). In addition, analytical-grade dimethyl sulfoxide (DMSO), used as a solvent in the bioassay, was purchased from DEAJUNG Chemical (Siheung, Gyeonggi, Korea). Reagents and solvents, including AChE from electric eel, acetylthiocholine iodide (ATCh), BChE from equine serum, *S-butyrylthiocholine* iodide (BTCh), 5,5′- dithiobis [2-nitrobenzoic acid] (DTNB), bovine serum albumin (BSA), d-(+)-glucose, d-(−)-fructose, berberine, quercetin, and aminoguanidine (AMG), were purchased from Sigma-Aldrich Co. (St. Louis, MO, USA). The BACE1 (β-secretase) FRET assay kit was purchased from Pan Vera Co. (Madison, WI, USA).

### 3.3. Extraction, Fractionation, and Isolation of H. diffusa

The aerial parts of *H. diffusa* (7.7 kg) were dried and powdered, and then extracted in methanol (20 L × 3) at room temperature. The total filtrate was concentrated to dryness at 50 °C to yield the MeOH extract (2256.32 g). It was suspended in distilled water and then partitioned sequentially in Hx, DCM, EA, and *n*-BuOH. The results yielded Hx (50.72 g), DCM (56.07 g), EA (10.59 g), *n*-BuOH (45.19 g), and water (86.50 g) fractions. Among these five fractions, the EA and *n*-BuOH fractions were found to be most potent in the four anti-Alzheimer disease model assays. Therefore, open column chromatography of these active fractions was performed repeatedly, and five compounds were obtained in each of the EA and *n*-BuOH fractions.

The chromatographic analysis of the EA fraction was done using Sephadex LH-20 column, with an elution gradient of 40% to 100% MeOH to give six sub-fractions. Separation of sub-fraction 3 using open column chromatography with MCI gel, with 50% to 100% MeOH solvent system, yielded seven fractions. Using ODS column chromatography with 50% MeOH solvent system, four sub-fractions were isolated from sub-fractions 3-4. Compound **1** (845.4 mg) was isolated from fraction 3-4-3 using Sephadex LH-20 with 50% EtOH. Furthermore, compound **3** (161.9 mg) was isolated from sub-fractions 3-5 using ODS column chromatography with 40% MeOH. In addition, separation of sub-fraction 1 using MCI gel column chromatography with 10% to 100% MeOH solvent system yielded seven fractions. Using ODS column chromatography with 10% to 100% MeOH, compound **4** (122.6 mg) was isolated from sub-fractions 1-2, while sub-fractions 1-5 were separated from compounds **6** (165 mg) and **7** (129.7 mg) by performing ODS column chromatography with 20% MeOH.

The *n*-BuOH fraction was first analyzed over silica gel using a solvent system of chloroform and MeOH (6:1→1:1) to separate the seven fractions. Sub-fraction 3 was separated on an MCI gel column chromatography with 50% to 100% MeOH to obtain fractions 3-1 to 3-8. Three sub-fractions were isolated from sub-fractions 3-7. Then, compound **2** (916.8 mg) was isolated from fractions 3-7-3 using ODS column chromatography with 50% to 100% MeOH. In addition, six sub-fractions were separated from sub-fractions 3-5 using MCI column chromatography with 20% to 100% MeOH to yield compound **5** (19 mg). Furthermore, compound **8** (166.6 mg) was isolated from sub-fraction 3-3 using ODS column chromatography with 20% to 100% MeOH, while sub-fraction 4 was subjected to MCI gel column chromatography using a 30% to 100% MeOH gradient elution solvent system, and sub-fractions 4-1 to 4-10 were obtained. Then, compound **9** (215.7 mg) was isolated from sub-fraction 4-1 using ODS gel column chromatography with 30% to 100% MeOH. Sub-fraction 4-9 was analyzed on an ODS gel column using a solvent system of 40% to 100% MeOH to obtain nine sub-fractions. Finally, compound **10** (69.1 mg) was isolated from sub-fraction 4-9-5 by performing ODS column chromatography with 25% MeOH.

### 3.4. Identification of Compounds Isolated from H. diffusa

#### 3.4.1. NMR

1D nuclear magnetic resonance (NMR) spectra were analyzed at 600 MHz (^1^H-NMR) and 150 MHz (^13^C-NMR) using a JNM-ECZ600R spectrometer (JEOL, Tokyo, Japan). Samples were dissolved in deuterated methanol (CD_3_OD) and dimethyl sulfoxide (DMSO-*d*_6_). Chemical shifts are presented as ppm (parts per million) on the δ scale and coupling constants (*J*) are presented in Hertz.

#### 3.4.2. UPLC-ESI/LTQ-Orbitrap-HRMS Conditions

The molecular weights of the compounds isolated from *H. diffusa* were confirmed using UPLC-ESI/LTQ-Orbitrap-HRMS. All samples were dissolved in distilled water. The column (Agilent ZORBRAX SB C_18_, 2.1 × 50 mm, 1.8 μm) and sampler temperatures were set to 30 °C and 15 °C, respectively. The mobile phase consisted of solvent A (0.1% formic acid in water) and solvent B (0.1% formic acid in acetonitrile). The gradient conditions were 0–18 min, 5–50% B; 18–20 min, 50–100% B. The flow rate was 0.3 mL/min, and the sample injection volume was 5.0 μL for the standard solution and 2.0 μL for the extract solution. The optimal analysis conditions were as follows: spray capillary voltage, 3.0 kV; S-lens RF level, 50.0 V; capillary temperature, 360 °C; heater temperature, 300 °C; sheath gas flow rate, 45 L/h; auxiliary gas flow rate, 10 L/h; full MS resolution, 35,000 (FWHM @ *m*/*z* 200); full MS AGC target, 3e^6^; and full MS maximum IT, 200 ms.

In case of UHPLC-ESI/QTOF-MS for identifying compounds **α** and **β**, the *H. diffusa* extract was dissolved in 70% MeOH. The column (Aquity CSH C_18_, 2.1 × 100 mm, 1.7 μm) and sampler temperatures were set to 30 °C and 15 °C, respectively. The mobile phase consisted of solvent A (10 mM ammonium acetate (pH 3.5) with formic acid) and solvent B (acetonitrile). The gradient conditions were 0–10 min, 10–30% B; 10–15 min, 30–65% B. The flow rate was 0.3 mL/min, and the sample injection volume was 5.0 μL. The optimal analysis conditions were as follows: spray capillary voltage, 2.0 kV; source and desolvation temperature, 100 & 500 °C; cone and desolvation gas, 0 and 700 L/h; acquisition range, *m*/*z* 50 to 1200; acquisition rate, 0.5 s; collision energy ramp, ramping 10 to 30 V, 20 to 40 V.

### 3.5. HPLC Analysis

To analyze the six bioactive components, such as *E*-6-*O*-*p*-coumaroyl scandoside methyl ester (**1**), *E*-6-*O*-feruloyl scandoside methyl ester (**3**), deacetylasperulosidic acid methyl ester (**4**), scandoside methyl ester (**8**), asperulosidic acid (**9**), and quercetin-3-*O*-[2″-*O*-(6‴-*O*-*E*-feruolyl)-β-d-glucopyranosyl]-β-d-glucopyranoside (**10**), from *H. diffusa*, the Waters Kromasil C_18_ column (4.6 × 250 mm, 5 μm) was used to analyze the major compounds isolated from *H. diffusa*. The mobile phase system consisted of 0.1% acetic acid in water (solvent A) and acetonitrile (solvent B) at a flow rate of 0.8 mL/min. The linear gradient elution was implemented with the following elution program: 0–40 min, 10–30% B; 40–50 min, 30–60% B. All eluents were filtered in a 0.45 μm PVDF syringe filter. The sample injection volume was 10 μL, and UV 254 nm was selected as the optimal wavelength for detecting the compounds. For preparation of extract stock solutions, plant powders were sonicated with 70% MeOH for 90 min and dried under vacuum by using a rotary evaporator at 50 °C. After then, they were dissolved in MeOH to a concentration of 10,000 ppm. Standard compound stock solutions were also dissolved in MeOH. Prior to injection, all analyzed stock solutions were strained using a 0.45 μm PVDF syringe filter. The standard calibration curve was constructed using five different concentrations. The linear relationship between peak area and concentration is described in Table 6. The concentrations of the six major components were calculated using regression equations based on the calibration curves.

### 3.6. Identification of Compounds Isolated from H. diffusa

#### 3.6.1. *E*-6-*O*-*p*-Coumaroyl Scandoside Methyl Ester (**1**)

C_26_H_30_O_13_; ESI/LTQ-Orbitrap-HRMS *m*/*z*: 549.1610 [M−H]^−^; ^1^H-NMR (CD_3_OD): 7.60 (1H, d, *J* = 7.8 Hz, H-7′), 7.56 (1H, s, H-3), 7.42 (2H, d, *J* = 9.0 Hz, H-2″,6″), 6.76 (2H, d, *J* = 8.4 Hz, H-3″,5″), 6.29 (1H, d, *J* = 15.6 Hz, H-8′), 5.80 (1H, m, H-7), 5.62 (1H, m, H-6), 5.26 (1H, d, *J* = 6.6 Hz, H-1), 4.65 (1H, d, *J* = 8.4 Hz, H-1′), 4.33 (1H, d, *J* = 15.1 Hz, H-10), 4.17 (1H, d, *J* = 15.7 Hz, H-10), 3.83 (1H, d, *J* = 11.7 Hz, H-6′), 3.59 (1H, m, H-6′), 3.59 (3H, s, H-12), 3.26–3.59 (4H, m, H-2′,3′,4′,5′), 3.17 (1H, m, H-9); ^13^C-NMR (CD_3_OD): 168.9 (C-11), 168.6 (C-9′), 161.0 (C-4′), 153.9 (C-3), 150.1 (C-8), 146.4 (C-7′), 131.0 (C-2′,6′), 127.1 (C-7,1′), 116.7 (C-3′,5′), 115.3 (C-8′), 109.8 (C-4), 100.2 (C-1″), 97.8 (C-1), 83.5 (C-6), 78.3 (C-3″), 77.8 (C-5″), 74.7 (C-2″), 71.4 (C-4″), 62.6 (C-6″), 60.9 (C-10), 52.0 (C-12), 46.9 (C-9), 42.3 (C-5).

#### 3.6.2. 6-*O*-*p*-Coumaroyl Scandoside Methyl Ester (**2**)

C_26_H_30_O_13_; ESI/LTQ-Orbitrap-HRMS *m*/*z*: 549.1609 [M−H]^−^; ^1^H-NMR (DMSO-*d*_6_): 7.57 (1H, d, *J* = 14.4 Hz, H-α), 7.56 (2H, d, *J* = 9.0 Hz, H-6″), 7.54 (2H, d, *J* = 9.0 Hz, H-2″), 7.46 (1H, s, H-3), 6.79 (2H, d, *J* = 9.0 Hz, H-3″,5″), 6.38 (1H, d, *J* = 16.2 Hz, H-β), 5.73 (1H, m, H-7), 5.28 (1H, d, *J* = 5.4 Hz, H-1), 3.57 (3H, s, -COOCH_3_); ^13^C-NMR (DMSO-*d*_6_): 166.6 (C-11), 166.2 (C-CO), 160.0 (C-4″), 152.5 (C-3), 150.1 (C-8), 144.8 (C-α), 130.4 (C-2″,6″), 125.2 (C-1″), 124.9 (C-7), 115.9 (C-3″,5″), 114.4 (C-β), 108.3 (C-4), 98.6 (C-1′), 95.3 (C-1), 81.6 (C-6), 77.4 (C-3′), 76.7 (C-5′), 73.3 (C-2′), 70.1 (C-4′), 61.2 (C-6′), 59.1 (C-10), 51.3 (C-12), 45.7 (C-9), 40.3 (C-5).

#### 3.6.3. *E*-6-*O*-Feruloyl Scandoside Methyl ester (**3**)

C_27_H_32_O_14_; ESI/LTQ-Orbitrap-HRMS *m*/*z*: 579.1732 [M−H]^−^; ^1^H-NMR (DMSO-*d*_6_): 7.53 (1H, d, *J* = 15.6 Hz, H-α), 7.41 (1H, s, H-3), 7.10 (1H, d, *J* = 1.2 Hz, H-2″), 7.08 (1H, dd, *J* = 1.2, 8.4 Hz, H-6″), 6.76 (1H, d, *J* = 8.4 Hz), 6.29 (1H, d, *J* = 15.6 Hz, H-β), 5.75 (1H, m, H-7), 5.58 (1H, m, H-6), 5.22 (1H, d, *J* = 6.6 Hz, H-1), 4.60 (1H, d, *J* = 7.8 Hz, H-1′), 4.30 (1H, d, *J* = 15.0 Hz, H-10), 4.13 (1H, d, *J* = 15.6 Hz, H-10), 3.79 (1H, s, -OMe), 3.78 (1H, d, *J* = 12.0 Hz, H-6′), 3.55 (1H, m, H-6′), 3.53 (1H, s, H-12), 3.24 (1H, d, *J* = 7.2 Hz, H-5), 3.24 (1H, m, H-3′), 3.17 (1H, m, H-5′), 3.12 (1H, m, H-4′), 3.11 (1H, m, H-2′), 2.96 (1H, dd, *J* = 4.2, 4.2 Hz, H-9); ^13^C-NMR (DMSO-*d*_6_): 166.5 (C-11), 166.1 (CO), 152.3 (C-3), 149.9 (C-8), 149.3 (C-3″), 147.9 (C-4″), 145.0 (C-α), 125.6 (C-1″), 124.7 (C-7), 123.1 (C-6″), 115.5 (C-5″), 114.6 (C-β), 111.2 (C-2″), 108.1 (C-4), 98.4 (C-1′), 95.1 (C-1), 81.4 (C-6), 77.3 (C-3′), 76.5 (C-5′), 73.2 (C-2′), 70.0 (C-4′), 61.0 (C-6′), 58.9 (C-10), 55.6 (-OMe), 51.1 (C-12), 45.6 (C-9), 40.1 (C-5).

#### 3.6.4. Deacetylasperulosidic Acid Methyl ester (**4**)

C_17_H_24_O_11_; ESI/LTQ-Orbitrap-HRMS *m*/*z*: 403.1252 [M−H]^−^; ^1^H-NMR (CD_3_OD): 7.63 (1H, d, *J* = 1.6 Hz, H-3), 5.99 (1H, d, *J* = 2.1 Hz, H-7), 5.04 (1H, d, *J* = 9.0 Hz, H-1), 4.70 (1H, d, *J* = 7.8 Hz, H-1′), 4.44 (1H, d, *J* = 16.2 Hz, H-10), 4.19 (1H, d, *J* = 15.6 Hz, H-10), 3.74 (3H, s, COCH_3_), 3.00 (1H, ddd, *J* = 6.6, 5.4, 1.2 Hz, H-5), 2.55 (1H, dd, *J* = 8.4, 7.8 Hz, H-9); ^13^C-NMR (CD_3_OD): 169.4 (C-11), 155.3 (C-3), 151.5 (C-8), 129.7 (C-7), 108.2 (C-4), 101.5 (C-1), 100.4 (C-1′), 78.5 (C-3′), 77.8 (C-5′), 75.3 (C-6), 74.9 (C-2′), 71.6 (C-4′), 62.8 (C-6′), 61.6 (C-10), 51.8 (COCH_3_), 45.8 (C-9), 42.6 (C-5).

#### 3.6.5. Asperuloside (**5**)

C_18_H_22_O_11_; ESI/LTQ-Orbitrap-HRMS *m*/*z*: 413.1093 [M−H]^−^; ^1^H-NMR (CD_3_OD): 7.29 (1H, d, *J* = 1.8 Hz, H-3), 5.95 (1H, s, H-7), 5.72 (1H, d, *J* = 2.2 Hz, H-1), 5.56 (1H, d, *J* = 6.0 Hz, H-6), 4.85 (1H, s, H-10), 4.68 (1H, d, *J* = 7.8 Hz, H-1′), 4.33–3.15 (1H, m, H-2′,3′,4′, and 5′), 3.90–3.65 (1H, m, H-6′), 3.64–3.17 (1H, m, H-5,9), 2.06 (1H, s, AcO); ^13^C-NMR (150 MHz, CD_3_OD): 172.5 (AcO), 172.2 (C-11), 150.2 (C-3,8), 129.1 (C-7), 106.1 (C-4), 99.9 (C-1), 93.2 (C-1′), 86.3 (C-6), 78.3 (C-3′), 77.8 (C-5′), 74.6 (C-2′), 71.5 (C-4′), 62.7 (C-6′), 61.9 (C-10), 45.2 (C-9), 37.4 (C-5).

#### 3.6.6. 6-*O*-Methyldeacetylasperulosidic Acid Methyl Ester (**6**)

C_18_H_26_O_11_; ESI/LTQ-Orbitrap-HRMS *m*/*z*: 417.1402 [M−H]^−^; ^1^H-NMR (CD_3_OD): 7.68 (1H, d, *J* = 1.2 Hz, H-3), 6.24 (1H, d, *J* = 2.4 Hz, H-7), 5.04 (1H, d, *J* = 9.0 Hz, H-1), 4.78 (1H, d, *J* = 7.8 Hz, H-1′), 4.55 (1H, dd, *J* = 14.4, 1.8 Hz, H-10), 4.53 (1H, ddd, *J* = 6.6, 1.8, 1.8 Hz, H-6), 4.28 (1H, d, *J* = 15.6 Hz, H-10), 3.90 (1H, dd, *J* = 12.0, 1.8 Hz, H-6′), 3.81 (3H, s, 11-COOMe), 3.74 (1H, dd, *J* = 12.6, 5.4 Hz, H-6′), 3.32 (3H, s, 6-OMe), 3.15 (1H, ddd, *J* = 7.2, 6.0, 1.2 Hz, H-5); ^13^C-NMR (CD_3_OD): 169.4 (C-11), 155.0 (C-3), 152.8 (C-8), 127.5 (C-7), 108.1 (C-4), 101.7 (C-1), 100.7 (C-1′), 84.9 (C-6), 78.2 (C-5′), 77.8 (C-3′), 74.9 (C-2′), 71.3 (C-4′), 62.4 (C-6′), 61.7 (C-10), 57.3 (6-OMe), 51.8 (11-COOMe), 45.9 (C-9), 42.0 (C-5).

#### 3.6.7. 6-*O*-Methylscandoside Methyl Ester (**7**)

C_18_H_26_O_11_; ESI/LTQ-Orbitrap-HRMS *m*/*z*: 417.1402 [M−H]^−^; ^1^H-NMR (CD_3_OD): 7.31 (1H, d, *J* = 0.7 Hz, H-3), 5.74 (1H, t, *J* = 2.0 Hz, H-7), 5.54 (1H, d, *J* = 3.6 Hz, H-1), 4.50 (1H, d, *J* = 7.8 Hz, H-1′), 4.19 (1H, dd, *J* = 15.0, 1.2 Hz, H-10), 4.11 (1H, s, H-6), 4.08 (1H, d, *J* = 15.4 Hz, H-10), 3.80 (1H, dd, *J* = 11.4, 1.8 Hz, H-6′), 3.62 (3H, s, 11-COOMe), 3.57 (1H, dd, *J* = 12.0, 6.0 Hz, H-6′), 3.34 (3H, s, 6-OMe), 3.20 (2H, m, H-5,9); ^13^C-NMR (CD_3_OD): 169.0 (C-11), 153.5 (C-3), 149.6 (C-8), 127.3 (C-7), 110.4 (C-4), 99.9 (C-1′), 95.1 (C-1), 89.9 (C-6), 78.3 (C-3′), 77.9 (C-5′), 74.6 (C-2′), 71.5 (C-4′), 62.7 (C-6′), 60.3 (C-10), 57.0 (6-OMe), 51.6 (11-COOMe), 47.4 (C-9), 39.0 (C-5).

#### 3.6.8. Scandoside Methyl Ester (**8**)

C_17_H_24_O_11_; ESI/LTQ-Orbitrap-HRMS *m*/*z*: 403.1249 [M−H]^−^; ^1^H-NMR (CD_3_OD): 7.50 (1H, d, *J* = 1.8 Hz, H-3), 5.79 (1H, t, *J* = 2.4 Hz, H-7), 5.19 (1H, d, *J* = 7.2 Hz, H-1), 4.66 (1H, d, *J* = 8.4 Hz, H-1′), 4.54 (1H, dd, *J* = 4.2, 1.8 Hz, H-6), 4.34 (1H, d, *J* = 15.6 Hz, H-10), 4.18 (1H, d, *J* = 15.6 Hz, H-10), 3.84 (1H, dd, *J* = 12.6, 1.2 Hz, H-6′), 3.74 (3H, s, COOMe), 3.64 (1H, dd, *J* = 12.6, 5.4 Hz, H-6′), 3.25–3.37 (4H, m, H-2′,3′,4′, and 5′), 3.02 (1H, t, *J* = 6.6 Hz, H-9), 2.99 (1H, ddd, *J* = 7.2, 4.8, 1.2 Hz, H-5); ^13^C-NMR (CD_3_OD): 170.2 (C-11), 153.8 (C-3), 147.5 (C-8), 130.0 (C-7), 110.7 (C-4), 100.2 (C-1′), 98.26 (C-1), 82.2 (C-6), 78.4 (C-5′), 77.8 (C-3′), 74.7 (C-2′), 71.5 (C-4′), 62.6 (C-6′), 61.0 (C-10), 52.0 (COOMe), 47.1 (C-9), 45.5 (C-5).

#### 3.6.9. Asperulosidic Acid (**9**)

C_18_H_24_O_12_; ESI/LTQ-Orbitrap-HRMS *m*/*z*: 431.1192 [M−H]^−^; ^1^H-NMR (CD_3_OD): 7.51 (1H, d, *J* = 1.0 Hz, H-3), 5.89 (1H, d, *J* = 1.0 Hz, H-7), 4.93 (1H, d, *J* = 9.0 Hz, H-1), 4.82 (1H, s, H-6), 4.74 (1H, d, *J* = 15.6 Hz, H-10), 4.66 (1H, d, *J* = 15.0 Hz, H-10), 4.60 (1H, d, *J* = 7.2 Hz, H-1′), 3.73 (1H, dd, *J* = 12.0, 1.8 Hz, H-6′), 3.50 (1H, dd, *J* = 12.0, 6.6 Hz, H-6′), 3.09–3.29 (1H, m, H-2′,3′,4′, and 5′), 2.89 (1H, t, *J* = 6.0 Hz, H-5), 2.50 (1H, t, *J* = 8.4 Hz, H-9), 1.96 (1H, s, Ac-Me); ^13^C-NMR (CD_3_OD): 172.5 (CO-AcO, C-11), 155.1 (C-3), 145.9 (C-8), 131.8 (C-7), 108.7 (C-4), 101.1 (C-1), 100.5 (C-1′), 78.5 (C-3′), 77.8 (C-5′), 75.4 (C-6), 74.9 (C-2′), 71.5 (C-4′), 63.8 (C-10), 62.9 (C-6′), 46.2 (C-9), 42.5 (C-5), 20.8 (Ac-Me).

#### 3.6.10. Quercetin-3-*O*-[2′′-*O*-(6′′′-*O*-*E*-feruolyl)-β-d-glucopyranosyl]-β-d-glucopyranoside (**10**)

C_37_H_38_O_20_; ESI/LTQ-Orbitrap-HRMS *m*/*z*: 803.2200 [M−H]^−^; ^1^H-NMR (CD_3_OD): 7.60 (1H, dd, *J* = 8.4, 2.4 Hz, H-6′), 7.57 (1H, d, *J* = 1.8 Hz, H-2′), 7.31 (1H, d, *J* = 15.6 Hz, H-α), 6.85 (1H, d, *J* = 8.4 Hz, H-5′), 6.77 (1H, d, *J* = 1.2 Hz, H-2‴), 6.69 (1H, dd, *J* = 8.4, 1.2 Hz, H-6‴), 6.62 (1H, d, *J* = 7.8 Hz, H-5‴), 6.08 (1H, d, *J* = 1.2 Hz, H-8), 6.02 (1H, d, *J* = 16.2 Hz, H-β), 5.99 (1H, d, *J* = 1.2 Hz, H-6), 5.18 (1H, d, *J* = 7.2 Hz, H-1″), 4.74 (1H, d, *J* = 7.8 Hz, H-1‴), 3.74 (3H, s, OMe); ^13^C-NMR (CD_3_OD): 179.7 (C-4), 168.9 (C-γ), 165.7 (C-7), 162.8 (C-5), 158.2 (C-9), 158.1 (C-2), 150.3 (C-4‴), 149.7 (C-4′), 149.0 (C-3‴), 146.7 (C-α), 145.9 (C-3′), 135.1 (C-3), 127.3 (C-1‴), 123.8 (C-6‴), 123.7 (C-6′), 122.9 (C-1′), 117.3 (C-5′), 116.2 (C-2′, 5‴), 114.8 (C-β), 111.1 (C-2‴), 106.0 (C-1‴), 105.6 (C-10), 101.0 (C-1″), 99.8 (C-6), 94.7 (C-8), 84.7 (C-2″), 78.1 (C-3″), 77.7 (C-5″), 77.6 (C-3‴), 76.0 (C-2‴), 75.7 (C-5‴), 71.8 (C-4″), 70.8 (C-4‴), 64.5 (C-6‴), 62.2 (C-6″), 56.2 (OMe).

### 3.7. Bioactivities Assay

#### 3.7.1. Measurement of ChE Enzyme Assay

The inhibitory activities of the test samples against ChE (AChE and BChE) were evaluated using the spectrophotometric method developed in a previous study [38]. ATCh and BTCh were used as substrates to measure the inhibitory activities of AChE and BChE, respectively. The assay mixture contained 0.1 M sodium phosphate buffer (pH 7.8), 0.3 U/mL AChE or BChE, 0.5 mM DTNB, 0.6 mM ATCh or BTCh, and the tested sample solution that were mixed and incubated for 15 min at room temperature. All tested samples and positive control (berberine) were dissolved in 10% analytical grade DMSO at five different final concentrations (10–500 μg/mL for extracts and fractions or 10–500 μM for isolated compounds). Reactions started on addition of 10 μL of DTNB and 10 μL of either ATCh or BTCh. The ChE inhibitory activity was monitored based on the formation of the yellow 5-thio-2-nitrobenzoate anion at 412 nm for 15 min that was due to the reaction of DTNB and thiocholine released from ATCh or BTCh. All reactions were measured in 96-well microplates and tested in triplicate. The percentage of inhibition (%) was estimated using the following formula: {(Ac − As)/Ac} × 100, where Ac is the enzyme activity without the test sample and As is the enzyme activity with the test sample.

#### 3.7.2. Measurement of BACE1 Enzyme Assay

The BACE1 enzyme assay was performed in accordance with the manufacturer’s recommended protocol, with minor modifications. Briefly, the assay mixture contained 1.0 U/mL of BACE1, 50 mM sodium acetate buffer (pH 4.5), the substrate (750 nM Rh-EVNLDAEFK-Quencher in 50 mM ammonium bicarbonate), and samples. All samples and positive control (quercetin) were dissolved in 10% analytical grade DMSO at five different final concentrations. The reaction mixture was incubated for 60 min at 25 °C in the dark. Prior to measurement, stop solution (2.5 M sodium acetate) was added to the assay mixture. The BACE1 enzyme assay was determined by measuring the proteolysis of two fluorophores (Rh-EVNLDAEFK-Quencher) to form a fluorescent donor (Rh-EVNL) that increased in fluorescence wavelengths at 530–545 nm (excitation) and 570–590 nm (emission), respectively. All reactions were measured in black 96-well microplates and tested in triplicate. The percentage of inhibition (%) was obtained using the following formula: [1 − (S_60_ − S_0_)/(C_60_ − C_0_)] × 100, where C_60_ is the fluorescence of the control after 60 min of incubation, C_0_ is the initial fluorescence of the control, S_60_ is the fluorescence of the tested sample after 60 min of incubation, and S_0_ is the initial fluorescence of the tested sample. BACE1 inhibitory activity assay of each sample was presented in terms of IC_50_, as calculated from the log dose inhibition curve.

#### 3.7.3. Measurement of Inhibition of Formation of the AGE

The inhibitory activity of the formation of AGE was measured with a spectrophotometric method developed previously, with slight modifications [39]. Briefly, the assay mixture contained 50 mM phosphate buffer (pH 7.4) with 0.02% sodium azide, 0.4 M fructose and glucose, bovine serum albumin (10 mg/mL), and the sample. Next, the assay mixture was incubated at 60 °C for 2 days. After incubation, 200 μL of the reaction product was measured at excitation and emission wavelengths of 350 and 450 nm, respectively. All samples and positive control (aminoguanidine) were dissolved in 10% analytical-grade DMSO at five different final concentrations. All reactions were measured in black 96-well microplates and tested in triplicate. The percentage of inhibition (%) of the formation of AGE was estimated using the following formula: {(Ac − As)/Ac} × 100, where Ac is the fluorescence of the control and As is the fluorescence of the sample. Inhibition of the formation of AGE of each sample was presented in terms of IC_50_, as calculated from the log dose-inhibition curve.

### 3.8. Statistical Analysis

All assays were conducted in triplicates. Data are presented as the mean ± standard deviation (SD) and determined using one-way analysis of variance to evaluate differences between the positive control and treatment sample groups. The data were considered statistically significant at *p* < 0.05.

## 4. Conclusions

In this study, we determined the inhibitory potential of MeOH extract and fractions, isolated from *H. diffusa,* against ChE (AChE and BChE), BACE1, and the formation of AGE. The EA and BuOH fractions exhibited the strongest inhibitory activities, hence, we isolated ten major bioactive compounds, nine iridoid glycosides (**1**–**9**) and one flavonol glycoside (**10**), from these fractions, in accordance with bioassay-guided isolation. They were identified using ^1^H-NMR, ^13^C-NMR, and ESI/LTQ-Orbitrap-HRMS techniques. Then, compounds **1**–**10** from *H. diffusa* were evaluated for their anti-AD potencies, by conducting inhibitory assays of AChE, BChE, BACE1, and the formation of AGE. Since compounds **1**, **3**, **4**, **8**, **9**, and **10** had potential anti-AD inhibitory activities, we developed and validated a method that quantified and analyzed these compounds. Compound **α** (asperuloside) had the lowest anti-AD activity in all assays. Previous studies have reported that compound **β** (quercetin-3-*O*-sophoroside) does not show effective neuroinflammation inhibitory activity for the treatment of AD [40]. Therefore, among the eight biomarkers (compounds **1**, **3**, **4**, **8**, **9**, **10**, **α**, and **β**) separated by simultaneous HPLC analysis, compounds **α** and **β** that were identified as asperuloside and quercetin-3-*O*-sophoroside, were not selected from the chromatograms. Using this developed simultaneous analysis method, these six marker compounds (compounds **1**, **3**, **4**, **8**, **9**, and **10**) were successfully quantified in collected *H. diffusa* and *H. corymbosa* samples under optimized and efficient solvent extraction conditions. Among the samples, the *H. corymbosa* Lamark sample could be mistook for *H. diffusa* (which is also a genus of flowering plant in the family Rubiaceae), owing to their similar external appearance. This study suggested that compounds **1** (*E*-6-*O*-*p*-coumaroyl scandoside methyl ester) and **3** (*E*-6-*O*-feruloyl scandoside methyl ester) could be identified as biomarkers to distinguish between *H. diffusa* and *H. corymbosa* using the developed HPLC analytical method. This study also proved that *H. diffusa* is a novel remedy for AD and could be a potential drug. In addition, the developed analytical HPLC method could be applied to various fields for quality control of *H. diffusa*. It is necessary to conduct further research on *H. diffusa* to confirm the results obtained in this study, including prospective clinical trials, and investigate the medicinal effects of the isolated compounds. Furthermore, the compounds isolated from *H. diffusa* may be valuable therapeutic agents for other diseases.

## Figures and Tables

**Table 1:** Identification of compounds **1**–**10** in *H. diffusa* via UPLC-ESI/LTQ-Orbitrap-HRMS analysis.

Compound No.	Rt (min)	Formula	Mass Mode	Theoretical Mass	Observed Mass	Mass Error (Da)	Mass Accuracy (ppm)
1	10.55	C_26_H_30_O_13_	Negative	549.1624	549.1610	0.0014	2.5
2	10.56	C_26_H_30_O_13_	Negative	549.1610	549.1609	0.0001	0.2
3	10.83	C_27_H_32_O_14_	Negative	579.1721	579.1732	0.0011	1.9
4	2.14	C_17_H_24_O_11_	Negative	403.1262	403.1252	0.0010	2.5
5	5.87	C_18_H_22_O_11_	Negative	413.1096	413.1093	0.0003	0.7
6	5.68	C_18_H_26_O_11_	Negative	417.1382	417.1402	0.0020	4.8
7	6.19	C_18_H_26_O_11_	Negative	417.1382	417.1402	0.0020	4.8
8	4.08	C_17_H_24_O_11_	Negative	403.1234	403.1249	0.0015	3.7
9	4.96	C_18_H_24_O_12_	Negative	431.1187	431.1192	0.0005	1.2
10	9.63	C_37_H_38_O_20_	Negative	801.1889	801.1929	0.0040	4.9

**Table 2:** Qualitative analysis of compounds **α** and **β** by UPLC/QTOF-MS.

	Compound α (5)	Compound β
Retention time (UV)	2.89	5.14
Retention time (MS)	2.96	5.21
Expected formula (as M)	C_18_H_22_O_11_ (MW 414.36)	C_27_H_30_O_17_ (MW 626.52)
Theoretical monoisotopic molecular weight of deprotonated form	413.1084	625.1405
[M−H]^−^	413.1148	625.1511
Identification of compound	Asperuloside	Quercetin-3-*O*-sophoroside

**Table 3:** IC_50_ (inhibitory activity) of the *H. diffusa* extract and fractions for cholinesterase (acetylcholinesterase (AChE) and butyrylcholinesterase (BChE)), β-site amyloid precursor protein cleaving enzyme 1 (BACE1), and the formation of advanced glycation end-product (AGE).

Sample	IC_50_^a^ (μg/mL)			
	AChE	BChE	BACE1	AGE Formation
Ext.	102.01 ± 4.90 ***	99.79 ± 4.52 ***	113.44 ± 9.04 **	347.55 ± 7.27 ***
Hx fr.	54.51 ± 2.07 ***	7.28 ± 1.04 **	ND ^e^	355.52 ± 9.47 ***
DCM fr.	132.95 ± 12.91 **	36.74 ± 4.91 **	56.60 ± 2.95 ***	>1000
EA fr.	25.98 ± 3.07 **	31.22 ± 0.90 ***	14.84 ± 1.24 ***	99.32 ± 1.31 ***
*n*-BuOH fr.	58.92 ± 4.08 **	1.15 ± 0.32 *	26.92 ± 3.48 ***	109.27 ± 5.76 ***
Water fr.	ND ^e^	ND ^e^	ND ^e^	636.05 ± 25.69 ***
Berberine ^b^	0.11 ± 0.1 *	0.40 ± 0.09 *	-	-
Quercetin ^c^	-	-	8.65 ± 0.25 ***	-
AMG ^d^	-	-	-	131.92 ± 11.24 ***

Data are expressed as the mean ± SD (*n* = 3); ^a^ IC_50_ was calculated from the least-squares regression line of the logarithmic concentrations plotted against the residual activity; ^b^ Berberine was used as a positive control of ChE inhibitory activity; ^c^ Quercetin was used as a positive control of BACE1 inhibitory activity; ^d^ AMG was used as a positive control for the inhibition of the formation of AGE; ^e^ ND: not detected; * states a significant difference from control; * *p* < 0.05, ** *p* < 0.005, *** *p* < 0.001.

**Table 4:** IC_50_ of the compounds **1**–**10** for cholinesterase (acetylcholinesterase (AChE) and butyrylcholinesterase (BChE)), β-site amyloid precursor protein cleaving enzyme 1 (BACE1), and the formation of advanced glycation end-product (AGE) inhibition.

Compound	IC_50_^a^ (μM)			
	AChE	BChE	BACE1	AGE Formation
**1**	304.18 ± 12.15 ***	98.96 ± 2.74 ***	63.33 ± 4.56 ***	104.89 ± 14.47 ***
**2**	297.84 ± 22.68 **	26.22 ± 1.76 **	120.81 ± 14.76 ***	380.78 ± 42.72 ***
**3**	96.84 ± 5.29 ***	116.09 ± 29.39 *	121.14 ± 11.86 ***	66.61 ± 11.86 ***
**4**	172.26 ± 20.55 **	17.59 ± 0.78 ***	>500	ND ^e^
**5**	258.81 ± 7.48 ***	>500	>500	>1000
**6**	81.06 ± 5.58 **	32.24 ± 2.80 **	>500	ND ^e^
**7**	ND ^e^	11.59 ± 0.68 **	>500	ND ^e^
**8**	157.68 ± 13.18 **	16.18 ± 2.05 **	>500	ND ^e^
**9**	68.34 ± 5.11 **	80.29 ± 19.76 *	>500	ND ^e^
**10**	46.22 ± 1.59 ***	13.77 ± 0.37 ***	4.49 ± 1.86 ***	2.71 ± 0.06 ***
Berberine ^b^	0.31 ± 0.01 ***	1.82 ± 0.33 *	-	-
Quercetin ^c^	-	-	23.58 ± 4.17 ***	-
AMG ^d^	-	-	-	108.85 ± 5.27 ***

Data are expressed as the mean ± SD (*n* = 3); ^a^ IC_50_ was calculated from the least-squares regression line of the logarithmic concentrations plotted against the residual activity; ^b^ Berberine was used as a positive control of ChE inhibitory activity; ^c^ Quercetin was used as a positive control of BACE1 inhibitory activity; ^d^ AMG was used as a positive control of the inhibition of the formation of AGE; ^e^ ND: not detected; * states a significant difference from control; * *p* < 0.05, ** *p* < 0.005, *** *p* < 0.001.

**Table 5:** Quantity of compounds **1**, **3**, **4**, **8**, **9**, and **10** with reference to various solvent compositions and extraction times.

**Solvent Composition**	**30% MeOH 60 min**	**50% MeOH 60 min**	**70% MeOH 60 min**	**100% MeOH 60 min**
Compound **1** (mg/g)	1.266 ± 0.013	2.870 ± 0.033	3.190 ± 0.008	3.494 ± 0.003
Compound **3** (mg/g)	0.214 ± 0.002	0.419 ± 0.003	0.411 ± 0.003	0.419 ± 0.010
Compound **4** (mg/g)	0.039 ± 0.001	0.040 ± 0.003	0.032 ± 0.001	0.026 ± 0.001
Compound **8** (mg/g)	0.353 ± 0.001	0.166 ± 0.005	0.145 ± 0.001	0.126 ± 0.001
Compound **9** (mg/g)	0.827 ± 0.008	1.006 ± 0.004	0.923 ± 0.012	0.581 ± 0.005
Compound **10** (mg/g)	0.248 ± 0.001	0.404 ± 0.003	0.333 ± 0.008	0.188 ± 0.001
**Solvent Composition**	**30% EtOH 60 min**	**50% EtOH 60 min**	**70% EtOH 60 min**	**100% EtOH 60 min**
Compound **1** (mg/g)	1.827 ± 0.032	2.488 ± 0.005	2.653 ± 0.022	2.607 ± 0.007
Compound **3** (mg/g)	0.281 ± 0.007	0.349 ± 0.002	0.356 ± 0.001	0.334 ± 0.001
Compound **4** (mg/g)	0.036 ± 0.002	0.020 ± 0.001	0.024 ± 0.001	0.018 ± 0.001
Compound **8** (mg/g)	0.243 ± 0.002	0.123 ± 0.002	0.105 ± 0.001	0.065 ± 0.001
Compound **9** (mg/g)	0.845 ± 0.004	0.798 ± 0.005	0.764 ± 0.001	0.261 ± 0.001
Compound **10** (mg/g)	0.310 ± 0.002	0.336 ± 0.002	0.331 ± 0.001	0.101 ± 0.001
**Solvent Composition**	**70% MeOH 30 min**	**70% MeOH 60 min**	**70% MeOH 90 min**	**70% MeOH 120 min**
Compound **1** (mg/g)	2.756 ± 0.029	2.224 ± 0.022	3.011 ± 0.016	2.288 ± 0.027
Compound **3** (mg/g)	0.350 ± 0.001	0.305 ± 0.002	0.412 ± 0.004	0.309 ± 0.002
Compound **4** (mg/g)	0.031 ± 0.003	0.016 ± 0.001	0.027 ± 0.001	0.018 ± 0.001
Compound **8** (mg/g)	0.132 ± 0.004	0.105 ± 0.001	0.141 ± 0.002	0.104 ± 0.001
Compound **9** (mg/g)	0.836 ± 0.004	0.719 ± 0.003	0.982 ± 0.009	0.696 ± 0.002
Compound **10** (mg/g)	0.294 ± 0.002	0.272 ± 0.004	0.430 ± 0.001	0.280 ± 0.001

Data are presented as mean ± SD (*n* = 3) in mg/g dried sample; MeOH: methanol; EtOH: ethanol.

**Table 6:** Linear relation between peak area and concentration (*n* = 3).

Compound Number	Rt (min)	Regression Equation	r^2^	Linear Range (μg/mL)	LOD (μg/mL)	LOQ (μg/mL)
**1**	34.20/36.19	y = 1518.5x − 13,274	0.9994	10–500	0.04	0.13
**3**	35.41/37.21	y = 1459.1x − 11,482	0.9998	10–500	0.19	0.58
**4**	7.48	y = 974.39x − 7002.9	0.9995	10–500	0.14	0.43
**8**	8.48	y = 898.19x − 3765.1	0.9998	10–500	0.41	1.25
**9**	10.87	y = 795.26x − 5237.4	0.9995	10–500	0.61	1.84
**10**	31.22	y = 3123.7x − 21,456	0.9998	10–500	0.12	0.36

In the regression equation y = ax + b, x refers to the concentration of the compound (μg/mL), y the peak area; r^2^: the correlation of the equation; Rt: retention time; LOD: limit of detection; LOQ: limit of quantification.
